# Control of Eu Oxidation State in Y_2_O_3−x_S_x_:Eu Thin-Film Phosphors Prepared by Atomic Layer Deposition: A Structural and Photoluminescence Study

**DOI:** 10.3390/ma13010093

**Published:** 2019-12-23

**Authors:** José Rosa, Jonas Deuermeier, Pekka J. Soininen, Markus Bosund, Zhen Zhu, Elvira Fortunato, Rodrigo Martins, Mutsumi Sugiyama, Saoussen Merdes

**Affiliations:** 1 Beneq Oy, Olarinluoma 9, FI-02200 Espoo, Finland; jose.rosa@beneq.com (J.R.); pekka.j.soininen@beneq.com (P.J.S.); markus.bosund@beneq.com (M.B.); zhen.zhu@beneq.com (Z.Z.); 2 i3N/CENIMAT, Department of Materials Science, Faculty of Sciences and Technology, Universidade NOVA de Lisboa and CEMOP/UNINOVA, Campus de Caparica, 2829-516 Caparica, Portugal; j.deuermeier@campus.fct.unl.pt (J.D.); emf@fct.unl.pt (E.F.); rfpm@fct.unl.pt (R.M.); 3 Department of Electrical Engineering, Tokyo University of Science, 2641 Yamazaki, Noda, Chiba 278-8510, Japan; mutsumi@rs.noda.tus.ac.jp

**Keywords:** Y_2_O_2_S:Eu, Eu oxidation state, phosphor, photoluminescence

## Abstract

Structural and photoluminescence studies were carried out on Eu-doped Y_2_O_3−x_S_x_ thin films grown by atomic layer deposition at 300 °C. (CH_3_Cp)_3_Y, H_2_O, and H_2_S were used as yttrium, oxygen, and sulfur precursors, respectively, while Eu(thd)_3_ was used as the europium precursor. The Eu oxidation state was controlled during the growth process by following the Eu(thd)_3_ pulse with either a H_2_S or O_3_ pulse. The Eu(thd)_3_/O_3_ pulse sequence led to photoluminescence emission above 550 nm, whereas the Eu(thd)_3_/H_2_S pulse sequence resulted in emission below 500 nm.

## 1. Introduction

The study of red- and blue-emitting phosphors has major importance in the luminescence field due to the need for a wider color gamut in current displays and an increase in the efficiency of optoelectronic devices. Most of the studies on luminescent materials are carried out using luminescent lanthanide ions [1,2]. This choice is supported by high-intensity sharp line emission, persistent phosphorescence, and good luminescence efficiency generated by the d–f and f–f transitions [3].

The emission of various colors can be generated by doping a semiconductor matrix with different lanthanide ions that act as luminescent centers [4]. The trivalent europium ion (Eu^3+^) is the main choice to achieve red color emission. The strong emission is due to the intensive ^5^D_0_ → ^7^F_2_ electronic transition which generates a wavelength of 610–630 nm, depending on the host semiconductor matrix [5,6]. Europium can also assume a divalent state of oxidation (Eu^2+^) and can act as an important luminescent center. It is known for its broad emission band between ultraviolet and red, where the dominant emission of Eu^2+^ ions is attributed to the ^4^f_6_^5^d_1_ → ^4^f_7_ transition [7]. Many papers have reported Eu^2+^-doped material systems such as fluorides [8], chlorides [9], bromides [10], oxides [11], selenides [12], iodides [13], nitrides [14], and sulfides [15]. Because Eu^2+^ is highly dependent on the environment, each material emits in a specific range [7].

Y_2_O_2_S:Eu is a well-known phosphor which has mainly been investigated as a host for Eu^3+^ in order to obtain red emission [5,16,17,18,19,20,21]. This is supported by the similar dimensions of Eu^3+^ and Y^3+^ ionic radii of 1.01 Å and 0.96 Å at room temperature, respectively [22]. Y_2_O_2_S:Eu^3+^ was first reported by Hardy as a substitution for yttrium orthovanadate due to its high emission efficiency [23]. Since then, it has been studied and used in luminescence applications such as light-emitting diodes [24] and field emission displays [4].

During the last decade, Y_2_O_2_S doped with europium was successfully grown by several techniques including pulsed laser deposition [16], hydrothermal method [5], sol–gel template method [19], and decomposition method [17]. However, when using these growth techniques, the europium dopant tends to oxidize to its trivalent form Eu^3+^, as the divalent Eu^2+^ is unlikely to exist in Y_2_O_2_S, according to energy level considerations. This results in red being the main emission color generated by the Y_2_O_2_S:Eu material.

In this work, it is shown that the oxidation state of Eu in the Y_2_O_3−x_S_x_ (YOS) matrix can be controlled through the doping configuration by using the atomic layer deposition (ALD) method. Thus, during the growth process, Eu atoms on the surface were deliberately exposed to either oxidizing O_3_ or reducing H_2_S gas in order to generate a trivalent or a divalent oxidation state of Eu, respectively. This led to either red or violet/blue color emission from Y_2_O_3−x_S_x_:Eu (YOS:Eu).

## 2. Materials and Methods

**Figure 1:**
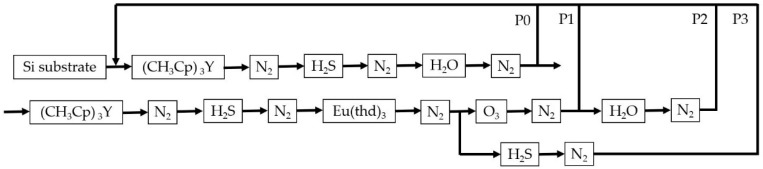
Schematic diagram of the processes used for the growth of the films.

Y_2_O_3−x_S_x_:Eu thin films were grown at 300 °C on (100)-oriented Si substrates using atomic layer deposition. The processes were carried out in a Beneq TFS-200 ALD-reactor (Beneq Oy, Espoo, Finland). (CH_3_Cp)_3_Y (98%, Intatrade, Anhalt-Bitterfeld Germany), H_2_O, and H_2_S were used as yttrium, oxygen, and sulfur precursors, respectively, whereas Eu(thd)_3_ (99.9%, Strem Chemicals, Kehl, Germany) was used as the Eu dopant precursor. N_2_ was used as the carrier and purging gas. Eu was introduced into the Y_2_O_3−x_S_x_ matrix in combination with either H_2_S or O_3_. During the processes, the pressure in the reactor was about 2 mbar. Process steps and parameters, including pulse sequences and pulse time, are summarized in Figure 1 and Table 1. Note that an N_2_ purge step was applied in all processes after each pulse. The purge time was 7 s.

The thickness of the grown films was determined with a SENTECH SE400adv ellipsometer (SENTECH Instruments GmbH, Berlin, Germany), using a 633 nm wavelength at 70° angle of incidence. The crystallinity was investigated by X-ray diffraction (XRD) using Cu Kα line in a Rigaku SmartLab (Rigaku Europe SE, Neu-Isenburg, Germany) high-resolution X-ray diffractometer. Chemical analyses were carried out by X-ray photoelectron spectroscopy (XPS). The spectra were measured with a Kratos Axis Supra spectrometer (Kratos Analytical Ltd., Manchester, UK), employing a monochromatic Al Kα source running at 225 W. The detailed spectra were either recorded at 10 eV pass energy for improved energy resolution or at 40 eV for faster acquisition (decreased X-ray exposure). Photoluminescence measurements were carried out at room temperature in a Perkin Elmer LS55 spectrophotometer (PerkinElmer Inc., Waltham, MA, USA), equipped with a pulsed Xenon discharge lamp of power equivalent to 20 kW for 8 μs duration. The samples were excited using two wavelengths, 266 and 355 nm.

## 3. Results

Figure 1 shows the schematic diagram of the ALD processes used for the growth of the Y_2_O_3−x_S_x_ and Y_2_O_3−x_S_x_:Eu thin films. Films with thicknesses between 50 and 300 nm were thus grown using the parameters summarized in Table 1. Thickness variations measured on about 50-nm-thick samples using ellipsometry were used to verify film homogeneity. Process P1, where the Eu(thd)_3_ pulse was followed by the O_3_ pulse, resulted in poor homogeneity with thickness fluctuations reaching 19%. The additional pulse of H_2_O after the O_3_ pulse in process P2 resulted in better thickness homogeneity with fluctuations down to about 10%. Due to the better film quality with process P2, in addition to processes P0 and P3, results related to films prepared with a combination of O_3_/H_2_O when introducing the Eu dopant in the matrix are presented. Therefore, in what follows, unless stated otherwise, the additional H_2_O pulse step is assumed when the Eu(thd)_3_/O_3_ pulse sequence related results are discussed.

### 3.1. Crystallinity

**Figure 2:**
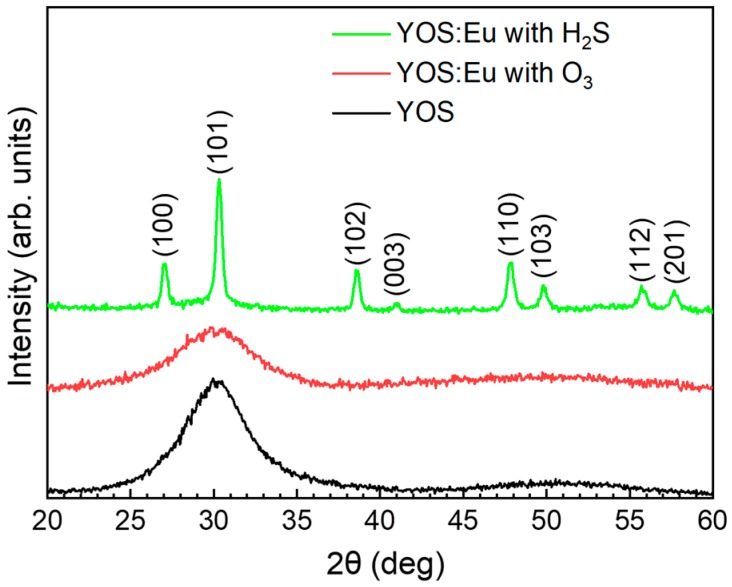
XRD of samples prepared using processes P0, P2, and P3.

Figure 2 shows XRD patterns measured between 20° and 60° at a fixed grazing incidence angle of 1° on samples grown using processes P0, P2, and P3. The undoped YOS sample and the Eu-doped one grown using the Eu(thd)_3_/O_3_ pulse sequence showed a single very broad peak around 30°, suggesting that the layers had a rather amorphous structure. However, the sample grown using the Eu(thd)_3_/H_2_S pulse sequence showed several sharp peaks, indicating that the film had a crystalline structure. Using JCPDS file no. 24-1424, Y_2_O_3−x_S_x_-related (100), (101), (102), (003), (110), (103), (112), and (201) reflections were identified, indicating that YOS:Eu grown using the Eu(thd)_3_/H_2_S pulse sequence had a hexagonal crystal structure with the lattice parameters a = 3.794 Å and c = 6.580 Å.

### 3.2. Chemical Analyses

An XPS overview spectrum (figure not shown) showed carbon, yttrium, oxygen, and sulfur in all the samples, as well as europium in the doped ones. Sputter cleaning was not carried out in order to avoid the introduction of surface carbon contamination into the layers. Charge accumulation took place due to the high resistivity of the material, which led to a shift of the peaks. The position of C, Y, O, and S core levels was corrected. A change in the Eu oxidation state from Eu^2+^ to Eu^3+^ during the measurement was also observed. Due to the uncertainty of the Eu oxidation state during the XPS measurements, spectra related to Eu core levels were not analyzed in detail. Nevertheless, Eu 3d core level spectra were used in the estimation of the elemental composition of the doped films.

**Figure 3:**
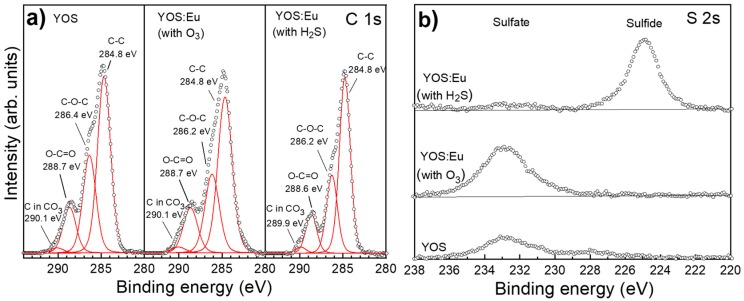
Measured and fitted (**a**) C 1s and (**b**) S 2s core level spectra. The films were prepared by processes P0, P2, and P3. Open symbols represent measured spectra, whereas red lines show fitting results.

Figure 3a shows measured and fitted XPS spectra for C 1s core levels in YOS and YOS:Eu films prepared by processes P0, P2, and P3. The spectra were deconvoluted by fitting the measured core level peaks with Voigt function. The corresponding binding energies are displayed in Figure 3a,b. C 1s core level spectra were composed of four peaks that were attributed mainly to CO_3_^2−^ and carbon contamination. In addition to the CO_3_^2−^ peak [25], all the samples showed C–C, C–O–C, and O–C=O bonds [25,26]. Figure 3b shows the S 2s core level spectra measured in the same samples. The spectra showed a significant difference between the sulfur bonds, depending on the growth process: while sulfate bonds (Y_2_(SO_4_)_3_) [27] were dominant in the YOS and YOS:Eu prepared using the Eu(thd)_3_/O_3_ sequence, sulfide bonds were identified only in the YOS:Eu film prepared using Eu(thd)_3_/H_2_S pulse sequence.

In Table 2, the elemental compositions of YOS:Eu samples, where Eu was introduced into the matrix in combination with either O_3_ (process P2) or H_2_S (process P3), are compared. C 1s, Eu 3d, O 1s, S 2s, and Y 3p core level spectra were used for the calculations. The film grown using the Eu(thd)_3_/O_3_ pulse sequence had a higher oxygen content than the one grown using the Eu(thd)_3_/H_2_S sequence, with an O/S ratio of 8.1 and 5.7, respectively. While the europium and yttrium contents were found to be lower in the sample prepared using O_3_, the carbon content was nearly the same in both films. Note that the carbon concentration measured by XPS was relatively high, with values exceeding 30%.

**Figure 4:**
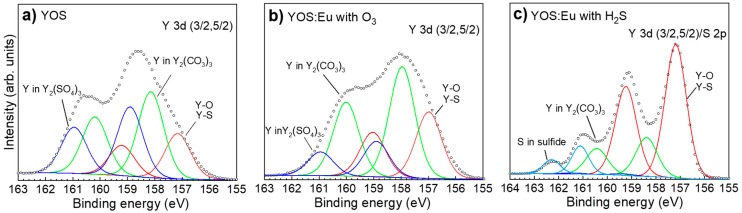
Measured and fitted Y 3d core level spectra in (**a**) undoped Y_2_O_3−x_S_x_ and (**b**) Y_2_O_3−x_S_x_:Eu prepared using the Eu(thd)_3_/O_3_ pulse sequence. (**c**) Measured and fitted Y 3d/S 2p spectra in Y_2_O_3−x_S_x_:Eu prepared using the Eu(thd)_3_/H_2_S pulse sequence. Open symbols represent measured spectra, whereas continuous lines show fitting results.

Figure 4a,b shows the measured and fitted XPS spectra for Y 3d core levels in YOS and YOS:Eu films prepared using the Eu(thd)_3_/O_3_ pulse sequence, respectively. Figure 4c shows the spectra for Y 3d together with S 2p core levels in YOS:Eu films prepared using the Eu(thd)_3_/H_2_S sequence. The measured spectra were fitted using Voigt function. Based on the results extracted from Figure 2, the following fitting restraints were applied:

- For Y 3d (5/2 and 3/2) doublets, a constant ratio of 3:2 and a constant separation distance of 2.05 eV were applied, whereas for S 2p (3/2 and 1/2) doublets, a constant ratio of 2:1 and a constant separation distance of 1.18 eV were applied.
- For YOS:Eu sample prepared using the Eu(thd)_3_/H_2_S pulse sequence, the sulfide 2p and the Y_2_(SO_4_)_3_ 3d peak area ratio were fixed to the values determined from the S 2s fit in Figure 2.
- Using the NIST (National Institute of Standards and Technology) XPS database [27], the Y 3d core level in Y_2_(SO_4_)_3_ was fixed to the range of 158.9–160 eV. Other binding energies (except the double splitting) were left to be adjusted automatically.
- The FWHM (Full Width at Half Maximum) of the Y 3d peaks was limited to 1.10 eV in order to keep the peaks uniform.

Note that, compared with other compounds, the concentration of Y_2_(SO_4_)_3_ was very low in the sample prepared using the Eu(thd)_3_/H_2_S pulse sequence. Therefore, Y_2_(SO_4_)_3_-related spectra are not visible in Figure 4c.

Table 3 summarizes the binding energies of fitted Y 3d and S 2p doublets deduced for the different samples. Based on the fitting results, the composition of the films was found to be strongly dependent on the deposition process, especially the doping configuration. The spectra were assigned to Y–O and/or Y–S bonds [25,27], Y_2_(CO_3_)_3_ [25], Y_2_(SO_4_)_3_ [27], and sulfur in sulfide form [27]. While Y–O/Y–S and Y_2_(CO_3_)_3_ were present in all films, S 2p doublets ranging between 160 and 164 eV were only found in the YOS:Eu sample grown using the Eu(thd)_3_/H_2_S sequence. A comparison of the relative amount of the identified yttrium compounds showed that the undoped YOS and the YOS:Eu prepared using the Eu(thd)_3_/O_3_ pulse sequence had the highest concentration of Y_2_(CO_3_)_3_, with relative amounts of 42.86% and 52.08%, respectively, while the YOS:Eu film prepared using the Eu(thd)_3_/H_2_S pulse had a relative Y_2_(CO_3_)_3_ amount of 16.23%.

### 3.3. Photoluminescence

**Figure 5:**
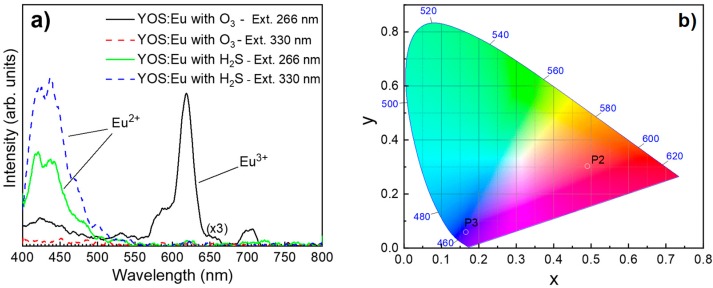
(**a**) Photoluminescence spectra measured from Y_2_O_3−x_S_x_:Eu samples prepared using either process P2 or P3. The measurements were carried out at room temperature. Excitation wavelengths of 266 and 330 nm were used. (**b**) CIE 1931 chromaticity diagram for Y_2_O_3−x_S_x_:Eu samples deduced from photoluminescence measurements in (**a**).

Figure 5a shows the photoluminescence spectra for YOS:Eu samples prepared using either the Eu(thd)_3_/O_3_ or the Eu(thd)_3_/H_2_S pulse sequence. The measurements were carried out at room temperature and both samples were excited with wavelengths of 266 and 330 nm. For an excitation wavelength of 330 nm, the Y_2_O_3−x_S_x_:Eu sample prepared using O_3_ showed no significant emission. However, for an excitation wavelength of 266 nm, luminescence spectra between 550 and 720 nm were obtained. The highest emission intensity was obtained at about 618 nm. Unlike the sample prepared using O_3_, for an excitation wavelength of 266 nm, the Y_2_O_3−x_S_x_:Eu sample prepared using H_2_S exhibited a dominant broad emission band below 500 nm. This band shape was dependent on the excitation wavelength. Thus, when the excitation wavelength was increased from 266 to 330 nm, the shoulder located at about 440 nm became more dominant than the one at about 420 nm. Figure 5b shows the 1931 CIE color coordinates deduced from the PL measurements using OriginLab Chromaticity Diagram script (Origin Pro 2019, Northampton, MA, USA). Thus, processes P2 and P3 led to the (x, y) values of (0.490, 0.303) and (0.165, 0.060), respectively.

## 4. Discussion

Y_2_O_3−x_S_x_:Eu thin films were successfully grown at 300 °C using ALD. The different characterization tools showed that the structural and emission properties of the obtained films depended strongly on the pulse sequences, especially the doping configuration. After each Eu(thd)_3_/O_3_ pulse sequence, the introduction of a subsequent H_2_O pulse improved the homogeneity of the films, as H_2_O is known to contribute to the increase of OH surface group concentration [28], most likely promoting the surface adsorption of yttrium species.

As expected, the films grown using the Eu(thd)_3_/O_3_ pulse sequence were found to have higher oxygen content in comparison with the ones grown using the Eu(thd)_3_/H_2_S sequence. However, the Eu concentration was lower in the films grown using O_3_, which indicates a much higher reactivity between surface Eu species and H_2_S, compared to O_3_. In addition to the Y_2_O_3−x_S_x_ phase, Y_2_(CO_3_)_3_ and either Y_2_(SO_4_)_3_ or sulfide compound were detected in all films, suggesting a rather complex film structure. The very high carbon concentration calculated from XPS spectra was due to Y_2_(CO_3_)_3_ and surface contamination, as no special precautions were taken to protect the film surface after the processes. Carbon contamination was most likely higher on the surface of YOS:Eu samples grown using the Eu(thd)_3_/H_2_S pulse sequence, considering their crystalline nature and therefore their larger surface area. Moreover, the concentration of carbonates measured by XPS also correlated with the crystalline structure of the films: unlike the highly polycrystalline structure of films grown using H_2_S, the undoped Y_2_O_3−x_S films as well as Y_2_O_3−x_S:Eu grown using O_3_ contained a large amount of yttrium carbonates. This is consistent with reports in which the crystallinity of Y_2_O_3_ thin films was improved by annealing the samples and therefore reducing the amount of carbonates in the films [29,30].

In addition to the Y–O/Y–S expected bonds, Y_2_(SO_4_)_3_ was identified in films grown using the Eu(thd)_3_/O_3_ sequence, whereas in the ones grown using the Eu(thd)_3_/H_2_S pulse sequence, S 2p doublets were found in the (160–164) eV energy range. The latter hints to the formation of a pure sulfide compound. It is therefore speculated that EuS species were most likely present in the films grown using the Eu(thd)_3_/H_2_S pulse sequence, considering that Eu atoms were deliberately exposed to the reducing H_2_S gas (i.e., H_2_S/Eu(thd)_3_/H_2_S pulse sequence) in order to generate a divalent oxidation state of Eu.

The samples were excited with energies (wavelengths of 266 and 330 nm) that were lower than the band gap of Y_2_O_2_S (E_g_ ≈ 5 eV). Therefore, the energy of the incident photons was too low to excite the YOS lattice. As a result, luminescent centers were excited directly. In the case of the Y_2_O_3−x_S_x_:Eu sample prepared using O_3_, for an excitation wavelength of 266 nm, the typical Eu^3+ 5^D_0_ → ^7^F_J_ (J = 0, 1, 2, 3, and 4) transitions were activated, resulting in red/pink emission spectra between 550 and 720 nm, the sharp and strong emission intensity of the ^5^D_0_ → ^7^F_2_ electronic transition being located at about 618 nm. For excitation wavelengths of both 266 and 330 nm, theY_2_O_3−x_S_x_:Eu sample prepared using H_2_S showed a single broad blue/violet emission below 500 nm that most likely originated from the 4f^6^5d → 4f^7^ electronic transitions of Eu^2+^ ions. The shape and intensity dependence of the Eu^2+^-related emission on the excitation wavelength, which has also been reported by other authors [31,32], suggests that the Eu^2+^ activator may be taking different kinds of ion sites in the host lattice [32].

## 5. Conclusions

In this work, Y_2_O_3−x_S_x_:Eu thin films were grown by atomic layer deposition. The crystallinity, composition, and emission properties of the obtained phosphors were correlated with the preparation conditions. XPS measurements showed the presence of Y_2_(CO_3_)_3_ and either Y_2_(SO_4_)_3_ or sulfide compounds, in addition to Y_2_O_3−x_S_x_. The use of Eu(thd)_3_ in combination with H_2_S led to films with a crystalline structure. It was shown that that the oxidation state of Eu in the host matrix could be successfully controlled through the doping configuration, and a violet/blue Y_2_O_3−x_S_x_:Eu phosphor was demonstrated. Red/pink emission was obtained when the Eu dopant was deliberately exposed to oxidizing O_3_, whereas a violet/blue emission was obtained when Eu was exposed to the reducing H_2_S gas.

## Figures and Tables

**Table 1:** Pulse sequences and corresponding pulsing times for Y_2_O_3−x_S_x_/Y_2_O_3−x_S_x_:Eu atomic layer deposition (ALD) processes.

Process	Pulse Sequence	Pulse Time (s)
P0	(CH_3_Cp)_3_Y/H_2_S/H_2_O	2.5/0.5/0.15
P1	(CH_3_Cp)_3_Y/H_2_S/H_2_O/(CH_3_Cp)_3_Y/H_2_S/Eu(thd)_3_/O_3_	2.5/0.5/0.15/2.5/0.5/2.5/3
P2	(CH_3_Cp)_3_Y/H_2_S/H_2_O/(CH_3_Cp)_3_Y/H_2_S/Eu(thd)_3_/O_3_/H_2_O	2.5/0.5/0.15/2.5/0.5/2.5/3/0.15
P3	(CH_3_Cp)_3_Y/H_2_S/H_2_O/(CH_3_Cp)_3_Y/H_2_S/Eu(thd)_3_/H_2_S	2.5/0.5/0.15/2.5/0.5/2.5/0.5

**Table 2:** Comparison of the elemental composition of Eu-doped Y_2_O_3−x_S_x_ films prepared using Eu(thd)_3_/O_3_ and Eu(thd)_3_/H_2_S pulse sequences.

Process	Y (at.%)	O (at.%)	S (at.%)	C (at.%)	Eu (at.%)
P2	10.6	48.7	6.0	30.8	3.9
P3	14.7	39.7	6.9	31.0	7.7

**Table 3:** Binding energy for Y 3d and S 2p doublets deduced for the compounds present in the Y_2_O_3−x_S_x_ and Y_2_O_3−x_S_x_:Eu films prepared by processes P0, P2, and P3.

Process	Y–O/Y–S		Y_2_(CO_3_)_3_		Y_2_(SO_4_)_3_		Sulfide	
	Y 3d 5/2 (eV)	Y 3d 3/2 (eV)	Y 3d 5/2 (eV)	Y 3d 3/2 (eV)	Y 3d 5/2 (eV)	Y 3d 3/2 (eV)	S 2p 3/2 (eV)	S 2p 1/2 (eV)
P0	157.16	159.21	158.14	160.19	158.90	160.95	-	-
P2	156.98	159.03	157.96	160.01	158.90	160.95	-	-
P3	157.18	159.23	158.38	160.43	159.30	161.35	161.11	162.29
